# Treatment and Prophylaxis of Post-pericardiotomy Syndrome in Cardiac Surgery Patients: a Systematic Review

**DOI:** 10.1007/s10557-021-07261-4

**Published:** 2021-09-21

**Authors:** Omar Giacinto, Alessandro Minati, Mario Lusini, Francesco Cardetta, Sara Saltarocchi, Mizar D’Abramo, Fabio Miraldi, Massimo Chello

**Affiliations:** 1 Department of Cardiovascular Surgery, University Campus Bio-Medico, Rome, Italy; 2 Department of Clinical, Internal Medicine, Anesthesiology and Cardiovascular Sciences, Sapienza University of Rome, Rome, Italy

**Keywords:** Post-pericardiotomy syndrome, Colchicine, Indomethacin, Dexamethasone

## Abstract

**Purpose:**

Post-pericardiotomy syndrome (PPS) is a common complication of cardiac surgery. This systematic review aimed to investigate the efficacy of colchicine, indomethacin, and dexamethasone in the treatment and prophylaxis of PPS.

**Methods:**

Literature research was carried out using PubMed. Studies investigating ≥ 10 patients with clinically PPS treated with colchicine, dexamethasone, and indomethacin and compared with placebo were included. Animal or in vitro experiments, studies on < 10 patients, case reports, congress reports, and review articles were excluded. Cochrane risk-of-bias tool for randomized trials (RoB2) was used for the quality assessment of studies.

**Results:**

Seven studies were included. Among studies with postoperative colchicine treatment, two of them demonstrated a significant reduction of PPS. In the single pre-surgery colchicine administration study, a decrease of PPS cases was registered. Indomethacin pre-surgery administration was linked to a reduction of PPS. No significant result emerged with preoperative dexamethasone intake.

**Conclusion:**

Better outcomes have been registered when colchicine and indomethacin were administered as primary prophylactic agents in preventing PPS and PE. Further RCT studies are needed to confirm these results.

## Introduction

Post-pericardiotomy syndrome (PPS) is a common complication of cardiac surgery that affects the pericardium and pleurae and is associated with significant morbidity and prolonged in-hospital stay. Although PPS pathophysiology remains unclear [1], surgical trauma and cardiopulmonary bypass (CPB) are reported as essential factors in triggering a systemic inflammatory response syndrome (SIRS) [2].

A single dose of long-acting corticosteroids is usually given during heart surgery to attenuate the inflammatory response, but definitive results of its efficacy are lacking [3].

Few days after surgery until the follow-up period, the pleuro-pericardial effusion may be detected, often complicating the postoperative course. In most cases, the accumulated fluid may cause respiratory distress (atelectasis) and infections, leading to a prolonged hospital stay or intensive care unit (ICU) admission [4]. Diagnosis of pericardial effusion relies on postoperative echocardiography, while pleural effusion (PE) is usually diagnosed through chest x-ray.

Although nonsteroidal anti-inflammatory drugs (NSAIDs), steroids, and colchicine are indicated as effective pharmacological agents to treat these conditions, side effects and secondary withdrawal of the medicaments could reduce their effectiveness [4–6]. To date, there is no scientific evidence of the resolution or prevention of PPS with a specific drug.

This systematic review aimed to investigate the role of current treatments in preventing or reducing cardiac surgery complications due to PPS or PE.

## Methods

The materials and methods were based on the PRISMA (Preferred Reporting Items for Systematic Reviews and Meta-Analysis) guidelines [7]. A systematic search was carried out on PubMed from September 2020 to April 2021 without time and language restrictions.

The components of the PICOS question were as follows: (patients) patients undergoing heart surgery; (intervention) colchicine, dexamethasone, or indomethacin; (comparison) placebo; (outcome) onset of PPS or PE; (study design) RCT.

The literature search strategy was based on the following keywords: (pericarditis OR pericardial effusion OR pleuro-pericardial syndrome) AND (colchicine OR dexamethasone OR indomethacin) AND (heart surgery).

## Study Selection and Selection Criteria

Studies on ≥ 10 patients with clinical pericarditis or post-pericardiotomy syndrome in which colchicine, dexamethasone, and indomethacin were compared with placebo were included. Publications referring only to animal or in vitro experiments, human studies on < 10 patients, case reports, congress reports, and review articles were excluded.

The complete list of articles obtained through the systematic search was scrutinized to remove duplicates and select the potentially relevant articles based on the title to answer the research question. Subsequently, the abstract screening was performed as well. The eligible studies were independently selected by two reviewers (OG and AM). From the remaining potentially relevant articles, those that met the inclusion and exclusion criteria were selected through full-text reading. Articles that did not meet inclusion criteria were not included in the present study.

The subsequent article selection was independently done by two authors (OG and AM). When there was disagreement, a third experienced reviewer was consulted to achieve a consensus.

The primary endpoint of the study was to investigate the efficacy of colchicine, dexamethasone, and indomethacin in the treatment and prevention of PPS and pleural effusion. The secondary endpoints were to assess the effectiveness of the three drugs in reducing hospital readmission, cardiac tamponade, symptom persistence, and atrial fibrillation after 72 h, as well as the safety and adverse effects of pharmacological treatment.

### Data Extraction

Two reviewers (OG and AM) independently extracted the data from the full texts of the studies that fulfilled the inclusion criteria. Disagreements were resolved through team discussions.

Data extraction was organized in tables that included the following information:

1. Study characteristics: first author, year, intervention arms, number of patients, randomization, and blinding
2. Patients’ clinical characteristics
3. Treatment indication and duration
4. Pharmacological dosage
5. Follow-up
6. Clinical outcomes

### Data Synthesis

All the data from the eligible articles were synthesized into a systematic summary. The characteristics of each study were reported. It was planned to synthesize a quantitative analysis (meta-analysis), but the methodology was not homogeneous among the included studies, and few studies were selected for indomethacin and dexamethasone.

### Risk of Bias Assessment

The quality of each RCT was independently assessed according to the Cochrane Risk of Bias Tool (RoB2) by two reviewers. Five domains of bias (i.e., randomization process, deviations from intended interventions, missing outcome data, measurement of the outcome, and selection of the reported results) were evaluated and reported. The Cochrane Handbook for Systematic Reviews of Interventions was used as a reference guide during the evaluation [8]. A judgment of “high” indicated a high risk of bias, “low” indicated a low risk of bias, and “some concerns” indicated the presence of bias due to lack of information or uncertainty about the potential for bias. Thus, the studies were categorized as having a low or high risk of bias or some concerns. Any discrepancy in the assessment of RoB2 was discussed to attain a consensus.

## Results

### Study Selection and Quality Assessment

**Fig. 1:**
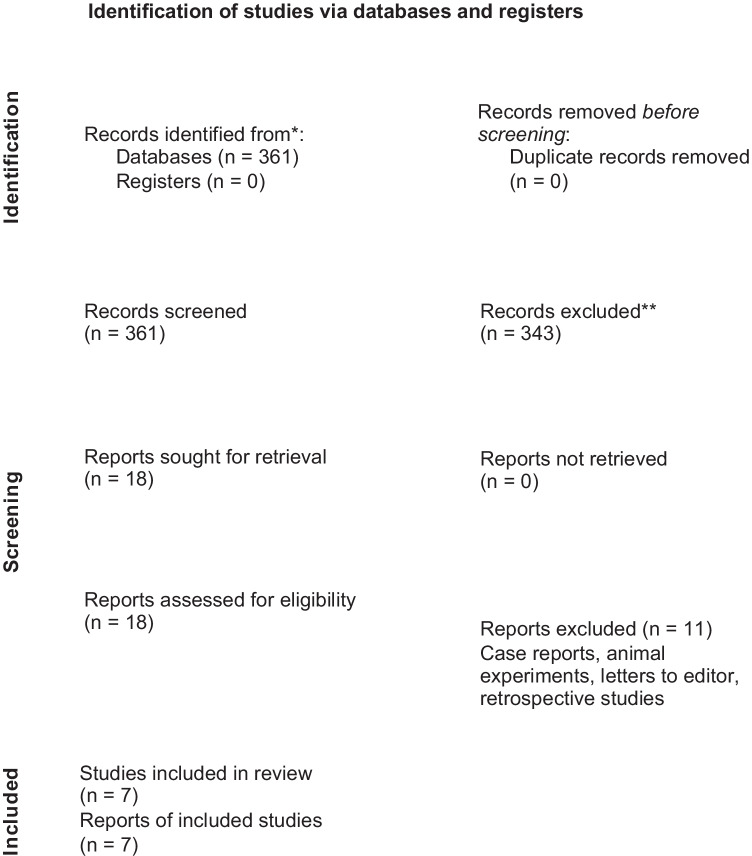
PRISMA flowchart

In this review, we examined an amount of 361 studies from 1977 to 2019. We included only seven randomized controlled trials [4, 9–14] (Fig. 1). Five trials investigated colchicine [4, 10–13], one indomethacin [9], and one dexamethasone [14]. Six were double-blind randomized trials [4, 9, 11–14], and one was a triple-blind randomized trial [10]. Five studies were multicenter trials [4, 11–14], while two studies were single-center randomized trials [9, 10]. Follow-up period varied from 1 to 12 months. The weight-adjusted colchicine dosage was 0.5 mg/kg in three studies [4, 11, 13] and 1 mg daily in two studies [10, 12].

The duration of colchicine treatment was between 14 and 30 days. It was started 48–72 h before surgery in one trial [11], on the 3^rd^ postoperative day in two trials [4, 13], on the 7^th^ day in another trial [10], and 30 days after heart surgery in one trial [12].

Indomethacin dosage was 25 mg 3 times/day for three days before surgery in one trial [9]. Treatment duration lasted for 6 weeks after the surgery.

Dexamethasone dosage was 1 mg/Kg i.v. as a single intraoperative bolus in one trial [14]. Studies characteristics are reported in Table 1.

**Table 1:** Studies’ characteristics

First author	Year	Design and setting	Indication	Sample	Age (years)	Male gender (%)	Treatment	Dose	Treatment duration (months)	Safety check	Follow-up (months)
Finkelstein et al. [13]	2002	Multicenter, randomized, double-blind	PPS Prevention	111	64 ± 11	73	Colchicine	On p.o. day 3 0.5 mg	PPS 3 months	Yes	3
Imazio et al. [4]	2010	Multicenter randomized, double-blind	PPS Prevention	360	65.7 ± 12.3	66	Colchicine	On p.o. day 3 1 mg followed by 0.5 mg twice daily	PPS 12 months	Yes	12
Inan et al. [9]	2011	Single-center randomized, double-blind	PE post heart surgery	85	50.3 ± 8.3 exp 47.8 ± 9.9 control	55	Indomethacin	25 mg × 3 daily beginning 7 days before surgery for six weeks p.o	PE 6 weeks		6 weeks
Bunge et al. [14]	2014	Multicenter randomizes double-blind	PPS prevention	822	65 ± 13.1 exp 64.7 ± 12.7 control	63	Dexamethasone	1 mg/kg single intraoperative dose	3 days		
Imazio et al. [11]	2014	Multicenter randomized, double-blind	PPS Prevention	360	67.5 ± 10.5	69	Colchicine	Starting 48 to 72 h before surgery 0,0.5 mg once/twice daily according to patient weight	PPS 3 months	Yes	3
Amoli et al. [10]	2015	Single-center randomized triple-blind	PE post heart surgery	149	57.4	60	Colchicine	1 mg on p.o 1 for 14 days	PPS 1 months	Yes	1
Meurin et al. [12]	2015	Multicenter randomized, double-blind	PE post-heart Surgery	197	64.5	86	Colchicine	1 mg/day for 14 days	PPS 14 days	Yes	6

Only five patients got lost during the follow-up of all studies. The groups did not differ in clinical characteristics and type of surgery. The seven prospective studies from 2002 to 2015 included 1,677 patients (mean age 60.8, male 67.4%). Baseline characteristics of treatment and control groups in all studies are reported in Table 2.

**Table 2:** Baseline studies’ characteristics

		Age	Male	BMI	HTN	DM	CABG	VD	Aorta	PE grade 1	PE grade 2	PE grade 3	PE grade 4
Amoli et al. (2015)	Placebo	59.3 ± 10.2	44 (59.5%)	27.2 ± 4.3	29 (39.2%)	26 (35.1%)	49 (66.2%)	8 (10.8%)		14 (18.9%)	20 (27%)	18 (24.3%)	4 (5.4%)
	Colchicine	55.3 ± 11.2	45 (60.0%)	26.5 ± 4.8	37 (49.3%)	19 (25.3%)	50 (66.7%)	15 (20%)		9 (12%)	35 (46.7%)	10 (13.3%)	5 (6.7%)
Bunge et al. (2014)	Placebo	64.7 ± 12.7	250 (62.3%)		192 (47.9%)	53 (13.1%)	140 (33%)	281 (66.7%)					
	Dexamethasone	65.5 ± 13.1	268 (63.7%)		211 (50.1%)	60 (14.3%)	117 (29%)	284 (70.8%)					
Finkelstein et al. (2002)	Placebo	65 ± 10	45 (40.5%)		48 (43,8%)	29 (26,6%)	52 (46.8%)	10 (-9%)					
	Colchicine	62 ± 12	36 (32.4%)		54 (48,9%)	31 (27,7%)	38 (-34%)	6 (5.4%)					
Imazio et al. (2010)	Placebo	67 ± 11	115 (64%)		125 (69.4%)	45 (25.0%)	77 (42.8%)	55 (30.6%)	8 (4.4%)				
	Colchicine	65 ± 14	124 (69%)		121 (67.2%)	39 (21.7%)	92 (51.1%)	51 (28.3%)	4 (2.2%)				
Imazio et al. (2014)	Placebo	68.0 ± 10.0	115 (63.9%)		122 (67.8%)	42 (23.3%)	59 (32.8%)	69 (38.3%)	11 (6.1%)				
	Colchicine	67.0 ± 11.1	133 (73.9%)		121 (67.2%)	38 (21.1%)	63 (35.0%)	62 (34.4%)	11 (6.1%)				
Inan et al. (2011)	Control	47.8 ± 9.9	24 (54%)		22 (50%)	9 (20%)		37 (84%)	44 (100%)				
	Indomethacin	50.3 ± 8.3	23 (56%)		23 (56%)	12 (29%)		33 (80%)	41(100%)				
Meuring et al. (2015)	Placebo	64.7 ± 10.6	88 (88.9%)	27.6 ± 4.3	62 (62.6%)	21 (21.2%)	52 (52.5%)	60 (60.7%)	15 (15.2%)	2.9 (3%)	35 (35.4%)	36 (36.4%)	28 (28.3%)
	Colchicine	64.2 ± 11.8	82 (83.7%)	26.7 ± 3.8	59 (60.2%)	23 (23.5%)	58 (59.2%)	48 (48.9%)	15 (15.3%)	3.0(3%)	27 (27.6%)	43 (43.9%)	28 (28.6%)

Legend: *VA* valvular disease; *PE* pleural effusion

**Fig. 2:**
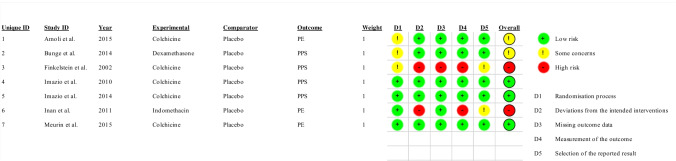
Risk of bias RoB2

Two studies were considered to have a “high” risk of bias [9, 13], two studies were considered to have “some concern” [10, 14], and three studies were considered to have a “low” risk of bias [11, 12, 15]. The risk of bias of each randomized clinical trial is reported in Fig. 2.

### Main Outcomes

Primary endpoints were related to registering the reduction of pericardial and pleural effusion in all the studies examined, while there was no homogeneity with secondary endpoints in the study groups.

Adverse effects contemplated gastrointestinal intolerance (GI), allergic reactions (AR), renal failure (RF), pancreatitis (PR), hepatotoxicity (HT), alopecia (AP), and leucopenia (LP) [4, 9–13]. Adverse effects ranged from 7 to 20% [4, 9–13].

Finkelstein et al. (2002), Imazio et al. (2010, 2014) study (all three studies referred to colchicine) and Inan et al. (2011) (indomethacin) reported as results a reduction of PPS and PE. On the contrary, Meurin et al. (2015), Amoli et al. (2015), and Bunge et al.’s study (2014) (dexamethasone) registered no reduction of PPS or PE.

#### Colchicine

Finkelstein et al. (2002) demonstrated a better outcome for patients undergoing colchicine treatment. PPS was diagnosed in 19 patients (17.1%), 5/47 cases (10.6%) in the colchicine group, and 14/64 (21.9%) in the placebo group, although without statistically significant difference (*p* = 0.135) [13]. Imazio et al. (2010) reported a significant reduction of the incidence of the PPS at 12 months in the colchicine group (*p* = 0.002) and in the secondary endpoints (hospitalization, cardiac tamponade, constrictive pericarditis, and relapses) at 18 months (*p* = 0.024). The rates of side effects and drug withdrawal were similar in the colchicine and placebo groups, although the colchicine group showed a trend towards an increased rate of both events. No severe side effects were recorded [4]. The same trend was confirmed in Imazio et al. (2014), where a significant reduction of incidence of PPS was also reported. PPS emerged in 35 patients (19.4%) assigned to colchicine group and 53 (29.4%) assigned to placebo group (absolute difference, 10.0%; 95% CI, 1.1–18.7%; number needed to treat = 10). There were no significant differences between the colchicine and placebo groups for the secondary endpoints of postoperative AF (colchicine: 61 patients [33.9%]; placebo: 75 patients [41.7%]; absolute difference: 7.8%; 95%CI, − 2.2 to 17.6%) or postoperative pericardial/pleural effusion (colchicine: 103 patients [57.2%]; placebo: 106 patients [58.9%]; absolute difference: 1.7%; 95%CI, − 8.5 to 11.7%), although there was a reduction in postoperative AF in the prespecified on-treatment analysis (placebo: 61/148 patients [41.2%]; colchicine: 38/141 patients [27.0%]; absolute difference: 14.2%; 95%CI, 3.3–24.7%). Adverse effects, mainly gastrointestinal events, were more frequently detected in the colchicine group [11].

Different results in the comparisons between colchicine and placebo were registered in Meurin et al. (2015) and Amoli et al. (2014), who reported no statistically significant difference between the two groups in the incidence of PPS (Meurin et al. *p* = 0.23; Amoli et al. *p* = 0.44). Mainly, Meurin et al. (2015) observed a similar decrease in mean pericardial effusion from baseline after treatment (− 1.1 ± 1.3 vs. − 1.3 ± 1.3 grades) in the colchicine and control group, respectively, without statistically significant mean difference between groups (− 0.19; 95% CI − 0.55 to 0.16, *p* = 0.23). In addition, no differences between colchicine and control groups were registered in the secondary endpoints in both studies [10, 12].

#### Indomethacin

Inan et al. (2011) explored the indomethacin effect on pericardial effusion when administered seven days before surgery and continued for six weeks after the operation. During hospitalization and follow-up, one patient in the indomethacin group and eight in the control group had significant pericardial effusion (*p* = 0.019) [9].

#### Dexamethasone

Bunge et al. (2014), who compared 1 mg/kg single dose intraoperative dexamethasone with the placebo group, reported no statistically significant difference between groups for primary prophylaxis on PPS. The incidence of PPS after drugs intake compared with placebo was 13.5% and 15.5% (relative risk 0.88, 95% CI 0.63–1.22). Complicated PPS had an incidence of 3.8% and 3.2%, respectively (relative risk 1.17, 95% CI 0.57–2). Also, for secondary endpoints, there was no statistical significance [14].

Complete results of primary and secondary endpoints are reported in Table 3.

**Table 3:** Primary and secondary endpoints

Authors	Primary endpoints			Secondary endpoints		
		Placebo	Treatment		Placebo	Treatment
Amoli et al. (2015)	Pretreatment PE variation	10.8 ± 2.5 mm	10.4 ± 2.4	Echocardiographic PE follow-up Mild	48%	28.6%
	Post-treatment PE variation	6.7 ± 6 mm	6.92 ± 5.5 mm	Echocardiographic PE follow-up Moderate		24.5%
				Echocardiographic PE follow-up Minimal		24.5%
Bunge et al. (2014)*	PPS	62 (15.5%)	57 (13.5%)	Complicated PPS	13 (3.2%)	16 (3.8%)
	Fever	64 (16%)	56 (13.3%)	Pericardiocentesis/thoracotomy for tamponade	8 (2%)	11(2.6%)
	Pericardial rubbing	87 (21.7%)	102 (24.2%)	Evacuation for PE	4 (1%)	5 (1.2%)
				Readmission for PPS	8 (2%)	3 (0.7%)
Finkelstein et al. (2002)	PPS	14 (21.9%)	5 (10.6%)	Pericarditis	17 (27%)	6 (12%)
Imazio et al. (2010)	PPS at 12 months	38 (21.1%)	16 (8.9%)	Recurrence	2 (1.1%)	0
	Fever 1 po week	7 (3.9%)	6 (3.3)	Cardiac tamponade	1 (0.6%)	0
	Pleuritic chest pain	23 (12.8%)	7 (3.9%)	Constrictive pericarditis	0	0
	Friction rub	15 (8.3%)	5 (2.7%)	PPS-related hospitalization	6 (3.3%)	1 (0.6%)
	Pleural effusion	46 (25.6%)	22 (12.2%)			
	New or worsening pericardial effusion	41 (22.8%)	23 (12.8%)			
Imazio et al. (2014)	PPS within 3 months	53 (29.4%)	35 (19.4%)	PO AF	75 (41.7%)	61 (33.9%)
				PO pericardial /pleural effusion	106 (58.9%)	103 (57.2%)
				Cardiac tamponade	3 (1.7%)	1 (0.6%)
				Pericardiocentesis /thoracentesis	13 (7.2%)	13 (7.2%)
				PPS recurrence	3 (1.7%)	3 (1.7%)
				Disease-related readmission	2 (1.1%)	2 (1.1%)
				Overall mortality	2 (1.1%)	6 (3.3%)
				Stroke	1 (0.6%)	2 (1.1%)
Inan et al. (2011)**	Plural effusion (0–14 days)	5 (11.3%)	0	Pleural effusion (2–6 weeks)	3 (6.8%)	1 (2.4%)
Meurin et al. (2015	Pericardial effusion	1.1 ± 1.3 cm	1.3 ± 1.3 cm	Tamponade	7 (7%)	6 (6%)
				Mean effusion width change from baseline	4.7 ± 6.9 mm	5.8 ± 6.6 mm
				Echocardiographic grade decrease	66.7%	74.5%
				AF	12.1%	15.3%

^*^Dexamethasone

^**^indomethacin

## Discussion

PPS presents with pleural or pericardial reaction, with symptomatology depending on the inflammatory response triggered after surgery. CBP time, surgical trauma, duration of the operation, and associated cytokine levels are thought to be essential factors in triggering a SIRS that leads to PPS [16–19].

The best management of pleural effusion is controversial. Clear indications on the right timing to perform a thoracentesis are lacking. Usually, a pleural effusion is complicated when thoracentesis is needed and is accompanied by alterations in clinical and arterial blood gas (ABG) parameters. Nonsteroidal anti-inflammatory drugs, colchicine, and steroids can be employed. Thoracentesis is typically performed as a final resort, considering that this procedure is not free from complications such as hemorrhage and pneumothorax. Pericardiocentesis is performed when the increased pressure in the pericardial cavity leads to echocardiographic signs of cardiac chambers compression (right and left atrial collapse, ventricular collapse, swinging heart, etc.) and subsequent hemodynamic instability.

In this systematic review, we identified seven randomized trials about the effectiveness of colchicine (5 studies), indomethacin (1 study), and dexamethasone (1 study) when used for primary prophylaxis purposes. The included studies had enrolled many patients, except for the indomethacin study [9], which was executed using a small sample. Finkelstein et al. (2002) performed a prospective, randomized, double-blind study. They demonstrated the statistical significance of the colchicine treatment in reducing PPS. No significant data were reported on specific secondary endpoints and adverse effects [13]. Imazio et al. (2010) demonstrated the efficacy of colchicine in reducing PPS events relating this reduction to pharmacological effects of colchicine on cells involved in the inflammation process. Colchicine may inhibit various leukocyte functions and has a preferential concentration in leukocytes [4].

Imazio et al. (2014) showed that preoperative administration of colchicine significantly reduced postoperative inflammation and its complication, especially PPS. As suggested by subgroup analysis, colchicine was more efficacious against C-reactive protein elevation. They avoided a loading dose and used weight-adjusted doses to obtain a higher degree of treatment adherence. However, high rates of adverse events were registered, such as gastrointestinal intolerance and drug discontinuation. These findings suggested that colchicine must be employed only in well-selected patients [11].

Meurin et al. (2015), assessing the effectiveness of colchicine to treat asymptomatic postoperative pericardial effusion, observed that the use of the colchicine did not significantly reduce the volume of PE [12]. The authors justified the absence of efficacy considering the inflammatory etiology not being the main trigger of PE, considering more likely aetiologies postoperative hemorrhagic effusion and PE secondary to heart failure. Nevertheless, they concluded that in patients with PPS, colchicine might be efficacious, as described in the ICAP study [15].

Izadi Amoli et al. (2015) performed a 2-week triple-blind trial comparing the effectiveness of colchicine in a high turnover specialized setting. The authors found no difference in the two groups regarding postoperative mild to moderate PE [10].

Two meta-analyses seem to agree with our findings of the efficacy of colchicine [20, 21].

Inan et al. (2011) investigated the prophylactic indomethacin effect on postoperative PE. Within the limitations of the low number of enrolled patients, the results of this study suggest that preoperative indomethacin intake may have a beneficial role on the outcome and incidence of postoperative PE. The authors’ conclusions indicated indomethacin as an alternative to other drugs to avoid postoperative PE [9].

Bunge et al. (2014) found no protective effect of a single high intraoperative dose of dexamethasone on PPS or complicated PPS in a cohort of patients undergoing valvular surgery [14].

## Conclusion

PPS and PE are the epiphenomena of the inflammatory status secondary to heart surgery, especially when CPB is adopted. Therefore, PPS and PE treatments on cardiac surgery are central concerns in avoiding complications such as cardiac tamponade or infections in the first postoperative days.

Limitations of the study were to be found in the heterogeneity of the population and the timing of drug administration. In the studies with postoperative colchicine administration, 2 of them demonstrated a significant reduction of PE. In the single pre-surgery colchicine administration study, a reduction of PE was obtained as well. In the indomethacin study, pre-surgery administration was linked to a reduction of PE. This result was not reported with pre-operatively dexamethasone intake. The included studies also demonstrated no reduction in PE of non-inflammatory etiology. In future studies, it could be helpful that this feature is excluded from primary endpoints.

Our findings showed a better outcome when colchicine and indomethacin were administered as primary prophylactic agents in reducing the risk of PPS and PE. Given their low adverse effects rates, clinical administration of colchicine and indomethacin in the preoperative setting could be considered an optimal solution to prevent PPS and PE.

## Data Availability

Available from the corresponding author upon request.
